# AKR1C3 overexpression may serve as a promising biomarker for prostate cancer progression

**DOI:** 10.1186/1746-1596-9-42

**Published:** 2014-02-26

**Authors:** Yuantong Tian, Lijing Zhao, Haitao Zhang, Xichun Liu, Lijuan Zhao, Xuejian Zhao, Yi Li, Jing Li

**Affiliations:** 1 College of Basic Medical Sciences, Jilin University, Changchun 130021, Jilin, China; 2 Tulane Cancer Center, Tulane University School of Medicine, 1430 Tulane Avenue SL-79, 70112 New Orleans, LA, USA; 3 Gannan Medical University, Ganzhou 341000, Jiangxi, China

**Keywords:** AKR1C3, Prostate cancer, Gleason score, PSA, Biomarker

## Abstract

**Background:**

Aldo-keto reductase family 1 member C3 (AKR1C3) is a key steroidogenic enzyme that is overexpressed in prostate cancer (PCa) and is associated with the development of castration-resistant prostate cancer (CRPC). The aim of this study was to investigate the correlation between the expression level of AKR1C3 and the progression of PCa.

**Methods:**

Sixty human prostate needle biopsy tissue specimens and ten LNCaP xenografts from intact or castrated male mice were included in the study. The relationship between the level of AKR1C3 expression by immunohistochemistry and evaluation factors for PCa progression, including prostate-specific antigen (PSA), Gleason score (GS) and age, were analyzed.

**Results:**

Low immunoreactivity of AKR1C3 was detected in normal prostate epithelium, benign prostatic hyperplasia (BPH) and prostatic intraepithelial neoplasia (PIN). Positive staining was gradually increased with an elevated GS in PCa epithelium and LNCaP xenografts in mice after castration. The Spearman’s r values (*r*_s_) of AKR1C3 to GS and PSA levels were 0.396 (*P* = 0.025) and -0.377 (*P* = 0.036), respectively, in PCa biopsies. The *r*_s_ of AKR1C3 to age was 0.76 (*P* = 0.011). No statistically significant difference was found with other variables.

**Conclusion:**

Our study suggests that the level of AKR.

1C3 expression is positively correlated with an elevated GS, indicating that AKR1C3 can serve as a promising biomarker for the progression of PCa.

**Virtual slides:**

The virtual slide(s) for this article can be found here: http://www.diagnosticpathology.diagnomx.eu/vs/7748245591110149.

## Background

Prostate cancer (PCa) is the second most frequently diagnosed cancer and the sixth leading cause of cancer-related mortality in men worldwide [1]. Androgen deprivation therapy (ADT) is a mainstay treatment for metastatic prostate cancer and is initially effective, with an 80-90% remission rate in patients and improved overall survival. However, most of the patients inevitably progress to CRPC [2,3]. Unfortunately, the median overall survival rate of CRPC is 23 to 37 months from the time of initiation of ADT [4]. Although the definitive mechanism underlying the progression of PCa remains poorly understood, two major mechanisms that result in the reactivation of the androgen axis in CRPC have been extensively studied [5]. One is the activation of the androgen receptor (AR)-mediated signaling pathway either by the amplification, overexpression or mutations of the AR [6,7]. The other mechanism mediates intratumoral androgen synthesis, involving either the *de novo* synthesis of AR ligands from cholesterol or the increased conversion of adrenal androgens (e.g., dehydroepiandrosterone or Δ^4^-Adione) to active androgens [8,9].

Based on the new theory of intratumoral androgen synthesis in prostate cancer cells, AKR1C3 was found to play a pivotal role in the synthesis of testosterone (T) and dihydrotestosterone (DHT), which are the most robust stimuli for activation of the growth, proliferation and metastasis of prostate cancer cells. In vitro experiments have shown that AKR1C3 is up-regulated in prostate cancer cells as a survival adaptation in response to T/DHT deprivation [9]. The overexpression of AKR1C3 was found to increase the intracellular synthesis of testosterone from 4-androstene-3,17-dione in LNCaP cells and resulted in resistance to the 5α-reductase inhibitor finasteride [10]. Dozmorov et al. demonstrated that the overexpression of AKR1C3 promotes angiogenesis and aggressiveness in PC-3 cells [11]. Several studies have reported low or undetectable levels of AKR1C3 in normal prostate epithelia, whereas elevated AKR1C3 levels have been found in localized, advanced or recurrent PCa and CRPC [12,13]. However, the correlation between the expression levels of AKR1C3 and the progression of PCa is unclear.

Recently, the benefit of prostate specific antigen (PSA) in the diagnosis and treatment of PCa in men was doubted by some researchers because PSA testing is associated with modest reductions in prostate cancer mortality, over diagnosis and over treatment [14]. Therefore, the next generation of PCa biomarkers that are superior to PSA or complement PSA testing should be explored. In our study, 60 human prostate needle biopsy tissue specimens and 10 murine tumor tissue specimens from intact or castrated male *nu*/*nu* mice were selected to detect AKR1C3 expression levels. The relationship between the levels of AKR1C3 expression and factors evaluated for PCa progression, including PSA, Gleason score (GS) and age, were analyzed, aiming to investigate whether AKR1C3 may serve as a potential biomarker for the progression of PCa.

## Materials and methods

### Patients and tissue samples

The PCa screening samples were obtained from 2001–2009 in the Prostate Diseases Prevention and Treatment Research Center of Jilin University in Changchun, Jilin province, China. None of the patients had previously undergone radical prostatectomy or other treatments, such as hormone or radiotherapy. In this study, 60 biopsies were selected for the assessment of AKR1C3 expression by immunohistochemistry staining. PCa case inclusion criteria were designated as follows: (1) detection of cancer within each prostate biopsy specimen, (2) GS of the biopsies equal to or greater than 6, and (3) adenocarcinoma specimens only. The clinicopathological features of PCa samples are summarized in Table 1. This study was approved by the Ethics Committee of Jilin University. The pathological diagnoses were determined by an experienced urological pathologist.

**Table 1:** Clinicopathological features of prostatic needle biopsy samples

**Sample**	**Case number**	**Mean age (Range)**	**Mean PSA (Range)**
BPH	10	64 (41–72)	13.7 (3.8-30.1)
PIN	10	84 (81–87)	18.1 (12.2-24)
GS = 6	10	69 (59–81)	52.7 (20.4-80.6)
GS = 7	10	69 (58–60)	57.9 (9.4-169)
GS = 8	10	70 (69–87)	59.8 (28–100)
GS = 9	10	70 (54–85)	71.2 (10–108.2)

### Cell culture and replication of LNCaP xenografts in mice

Human prostate LNCaP cells were obtained from the American Type Culture Collection at Passage 4. LNCaP cells were maintained in RPMI 1640 medium supplemented with 10% FBS, 2 mmol/L glutamine, 100 Units/mL penicillin and 100 μg/mL streptomycin. LNCaP cells (4 × 10^6^) were collected in 70 μL PBS and mixed with 70 μL Matrigel Matrix (Becton Dickinson Biosciences). The mixture was injected subcutaneously on one side of the dorsal flank of 6- to 7-week-old male *nu*/*nu* mice (National Cancer Institute, Frederick, MD). When the tumor volumes reached 100 mm^3^, the mice were randomized into a sham-operated group (n = 5) and a castrated group (n = 5). Briefly, after the intramuscular injection of general anesthesia with ketamine and xylazine (8.7 mg/100 g and 1.3 mg/100 g body weight, respectively) and the application of 75% alcohol to disinfect the scrotum, a small midline incision was made to expose the testes. The spermatic vessels were tied with 4.0 silk sutures, and the testes were removed. The incision was then closed using 4.0 silk sutures. In sham-operated mice, the skin of the scrotum was incised to expose the testes, followed only by closure of the incision using sutures. The animals were sacrificed at 3 weeks after the initial operation.

### Antibodies

Primary antibodies included AKR1C3 (NP6.G6.A6, mouse monoclonal antibody, 1:150, Abcam) and β-actin (mouse polyclonal antibody, 1:1000, Santa Cruz). Secondary antibodies were anti-mouse IgG (1:2000, Proteintech Group).

### Immunohistochemistry

Prostate tissue specimens were cut into approximately 4–6-μm-thick sections, mounted and baked at 55°C overnight. The sections were deparaffinized with xylene and re-hydrated in graded ethanol. Endogenous peroxidase activity was blocked by incubating the slides with 0.5% H_2_O_2_ in methanol for 10 min. Antigen retrieval was performed by heating the slides in 10 mM citric acid buffer (pH 6.0) at 121°C for 15 min in an autoclave. The slides were then washed with 0.1 M Tris–HCl at pH 7.6 (Tris) for 5 min and then incubated with Tris containing 10% goat serum to block non-specific binding. Next, the slides were incubated with AKR1C3 mAb (NP6.G6.A6, mouse monoclonal antibody, 1:150, Abcam) at a dilution of 1:200 at 4°C overnight. After washing with Tris, the slides were incubated with biotinylated goat anti-mouse secondary antibody (1:2000, Proteintech Group) in Tris containing 10% goat serum at room temperature for 1 h. Following the washes with Tris, HRP-conjugated streptavidin diluted in Tris containing 10% goat serum was added to the slides, which were incubated at room temperature for an additional 40 min. After a 10-min wash in Tris, a DAB-H_2_O_2_ substrate was added to the slides and incubated at room temperature for 6 min. The slides were then washed with distilled water and counterstained with hematoxylin. Next, the slides were dehydrated and sealed with Permount Mounting Media for subsequent visualization. The negative controls were handled in the same way except that PBS was applied in place of primary antibody.

AKR1C3 positive-staining exhibits a brown cytoplasmic and/or nuclear stain. Images of AKR1C3-positive cells were acquired from five randomly chosen fields (400×, magnification) per tissue section. The positive cell density was assessed using Image-Pro Plus 6.0 software (Media Cybernetics, Bethesda, MD, United States), and the results are presented as mean optical density (MOD) values. The negative controls were handled in the same way except that PBS was applied in place of a primary antibody.

### Statistical analyses

All of the results were analyzed using SPSS software, version 19.0 for Windows (SPSS Inc., IL, USA). One-way ANOVA was used to examine mean differences between groups. The data were recorded as the mean values ± standard deviation (SD). The Spearman’s Rho was applied to test for significant correlations between the variables. *P* values < 0.05 were considered as statistically significant.

## Results

### The expression of AKR1C3 in human prostate needle biopsy samples

**Figure 1:**
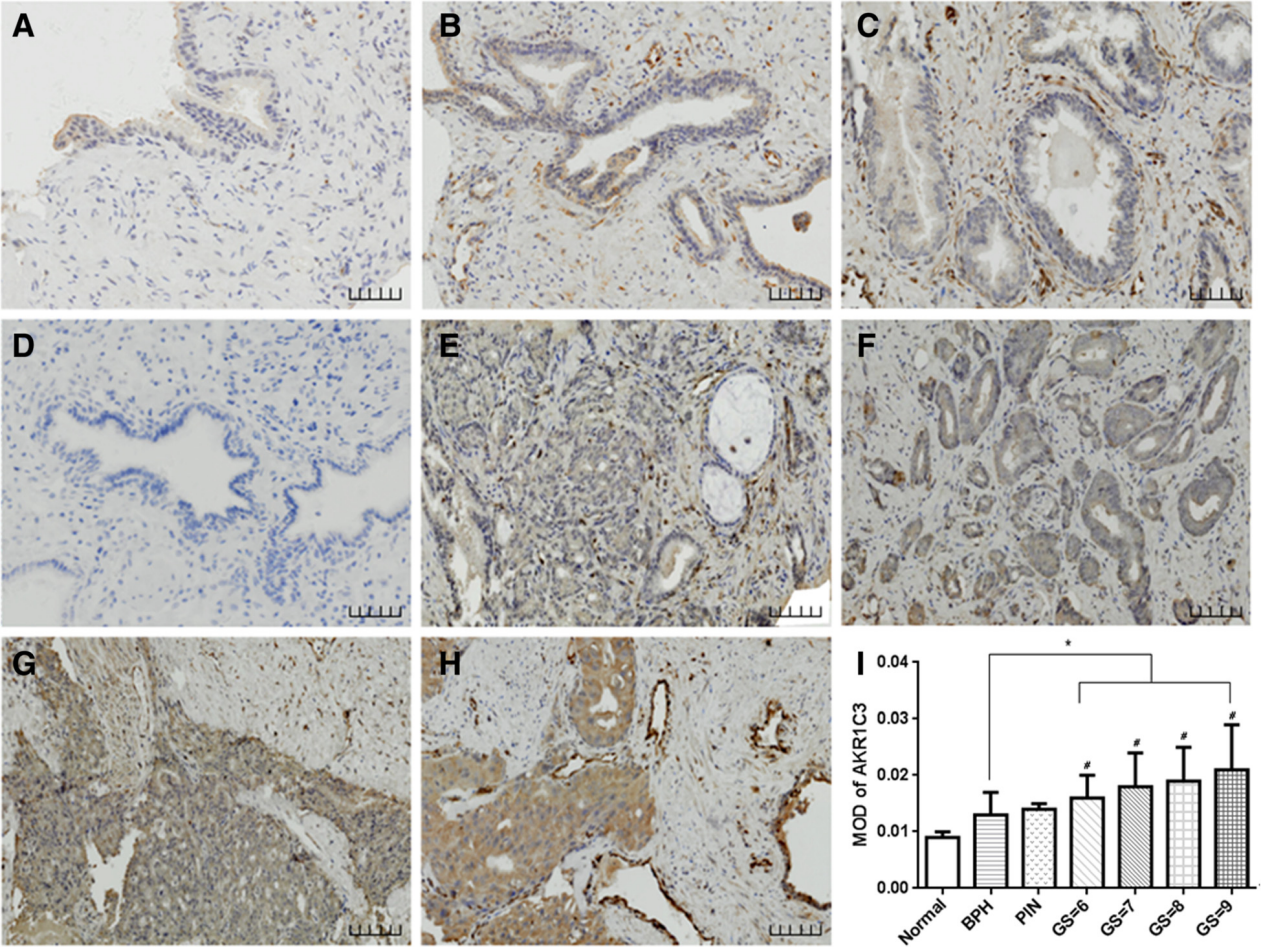
**Immunohistochemical staining of AKR1C3 in human prostatic needle biopsies. (A)** Normal prostate. **(B)** BPH. **(C)** PIN. **(D)** PBS negative control of prostate cancer tissue. **(E)**-**(H)** Different GS prostate cancer tissues. **(E)** Gleason score = 6. **(F)** Gleason score = 7. **(G)** Gleason score = 8. **(H)** Gleason score = 9. **(I)** The quantification bar for the MOD of AKR1C3. *P < 0.05, compared with BPH. ^#^*P* < 0.05, compared with PIN. Scale bar is 50 μm.

In this study, immunohistochemical staining for AKR1C3 in human prostate needle biopsy tissue specimens, including BPH, PIN and PCa, was carried out. Tissue sections that are representative of the immunohistochemical staining with monoclonal anti-AKR1C3 antibody are shown in Figure 1. In contrast to the negative staining observed in the normal glandular epithelium specimen (characterized by the small nuclei, Figure 1A) and the PBS control (Figure 1D), a few disseminated cells with brown positive immunoreactivity were visualized in the BPH (Figure 1B) as well as in the PIN samples (Figure 1C). Positive cytoplasmic staining was widely observed in the prostate cancer cells (characterized by the larger nuclei) (Figure 1E-H). The distribution of AKR1C3 expression is different between BPH and PCa. For BPH and PIN specimens, positive expression of AKR1C3 was observed in the stromal cells other than the epithelial cells, and for malignant prostate cancer specimens with GS greater than 6, a gradually stronger positive staining of AKR1C3 was detected in prostate cancer epithelial cytoplasm. The data also showed that AKR1C3 expression gradually increased with increasing GS (*r*_*s*_ = 0.396, *P* = 0.025) and slightly increased with age in BPH (*r*_*s*_ = 0.76, *P* = 0.011), but the MOD for positive AKR1C3 expression in prostate tumor tissues was significantly higher than that of the BPH specimens. This result implicated that the levels of AKR1C3 are closely associated with the PCa and GS.

### The expression of AKR1C3 in castrated mouse prostate cancer models

**Figure 2:**
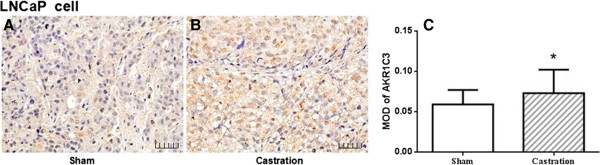
**Immunohistochemical expression of AKR1C3 in LNCaP xenograft mouse models with or without castration. (A)** LNCaP-sham. **(B)** LNCaP + castration. **(C)** MOD of AKR1C3 in LNCaP xenograft mouse models. *P < 0.05, compared with sham group. Scale bar is 50 μm.

To further confirm the relationship between the progression of PCa and the expression of AKR1C3, the LNCaP mouse model with or without castration was replicated. The aim of castration was to remove the circulating androgens, and the subcutaneous xenografts recurred at 3 weeks after castration, which reflected the state of prostate cancer progression to CRPC. The LNCaP prostate cancer cell line is androgen-dependent, and the expression levels of AKR1C3 in LNCaP tumors at 3 weeks after castration were significantly increased compared to those of the LNCaP sham tumors with MOD values of 0.081 ± 0.016 to 0.060 ± 0.018 (Figure 2A-C). These results indicate that androgen ablation likely stimulates AKR1C3 gene activation and might be attributed to prostate cancer progression.

### The correlation of AKR1C3 expression with clinicopathological features in prostate biopsy samples

In classical Partin tables, GS, PSA and age are key parameters for evaluating the progression of prostate cancer. The correlations of mean AKR1C3 expression with GS, mean PSA and age were analyzed and delineated (Tables 2 and 3). The data showed that AKR1C3 expression gradually increased with increasing GS, as indicated by the MOD, exhibiting a positive correlation (*r*_*s*_ = 0.396, *P* = 0.025). PSA is considered to be a putative maker for PCa progression and recurrence. The Spearman’s *r* values for PSA with the GS or AKR1C3 were analyzed. Serum PSA levels are not correlated with AKR1C3 (*r*_*s*_ = -0.016, *P* = 0.979) in BPH but are negatively correlated with AKR1C3 expression (*r*_*s*_ = -0.377, *P* = 0.036), which indicates that AKR1C3 is a better marker to reflect the clinicopathological stage and evaluation of PCa progression in those patients with low levels of PSA.

**Table 2:** AKR1C3 expression and clinicopathological features in PCa and BPH samples

**Variables**		**Cases of PCa**				**BPH**
		**GS = 6**	**GS = 7**	**GS = 8**	**GS = 9**	
Age (years)	<60	2	2	0	3	3
	61-75	6	4	6	4	5
	>75	2	4	4	4	3
MOD of AKR1C3	<0.017	4	3	2	2	9
	0.017-0.021	5	5	4	2	1
	>0.021	1	2	4	6	0
Serum PSA	<40 ng/ml	3	5	6	4	10
	>40 ng/ml	7	5	4	6	0

**Table 3:** Correlation of AKR1C3 expression with Gleason score, PSA and age

**Variable**		**PCa**			**BPH**	
		**GS**	**PSA**	**Age**	**PSA**	**Age**
	Spearman’s rho	0.396^*^	-0.377^*^	0.169	-0.016	0.760^*^
AKR1C3	sig (double)	0.025	0.036	0.356	0.979	0.011
	Number	40	40	40	10	10

*Spearman’s Rho test with significance of *P* < 0.05.

## Discussion

Androgens are known to play important roles in the pathogenesis of PCa [15]. Recently, the intratumoral synthesis of androgen from cholesterol or the conversion of adrenal precursor androgens to active androgens represent two important mechanisms underlying the progression of PCa and CRPC [5,6,8,13]. Several studies have indicated that AKR1C3 overexpression increases with PCa progression through the mechanisms underlying the key steroidogenic enzyme AKR1C3, which possesses 17β-hydroxysteroid dehydrogenase type 5 (17β-HSD5) activity, and PGF synthesis enzyme [13,16,17]. However, the correlation between the quantification of AKR1C3 expression and the progression of PCa is unclear.

In our study, AKR1C3 expression was investigated by immunohistochemical staining of prostate biopsy sections with different GSs. We found that AKR1C3 expression gradually increased with an elevated GS (*r*_*s*_ = 0.396, *P* = 0.025), implicating that AKR1C3 overexpression is closely associated with PCa malignancy. Interestingly, the distribution of AKR1C3 expression is different in PCa and preneoplastic change. For BPH and PIN specimens, most of the positive expression of AKR1C3 was observed in the stromal cells other than the epithelial cells; however, a gradually stronger positive staining of AKR1C3 was detected in the epithelial cells for malignant PCa specimens with GSs greater than 6. It is known that the epithelial cells in normal prostate are dependent on stromal cells secreting EGF, fibroblast growth factor (FGF), nerve growth factor (NGF) and IGF to support their growth and differentiation [18]. During malignant transformation of prostatic epithelial cells, androgen regulation shifts from paracrine to autocrine and prostatic epithelial cells adaptively acquire the intratumoral androgen synthesis ability to maintain the growth of tumor cells. It is reported that AKR1C3 is a pivotal enzyme in converting Δ4-dione to testosterone [13], 5α-DHT to 3α-diol [7], and androstenedione and dehydroepiandrosterone (DHEA) to intraprostatic testosterone in the progression of PCa and CRPC. Some studies showed that AKR1C3 has a preference in prostate cancer for the androstenedione to DHT by an alternative pathway [19]. Moreover, AKR1C3 possesses 11-ketoprostaglandin reductase activity and is capable of converting PGD2 to 9α, 11β-PGF2α, which promotes prostate cell proliferation through the PI3K/Akt signaling pathway in androgen receptor-negative PCa [11,20]. These data indicate that overexpression of AKR1C3 is the adaptive change that maintains tumor cell development and progression, and the consistency of AKR1C3 expression with the GS and higher expression in LNCaP xenografts of castrated mice in our study further strengthen the potential of AKR1C3 as a biomarker of PCa progression.

Recently, the potential prostate cancer biomarkers, such as prostate cancer antigen 3 (PCA3), TMPRSS2-ERG gene fusions and p501s (prostein), were investigated as auxiliary diagnosis candidates for prostate cancer [21-24]. Previous studies showed that poorly differentiated PCa tumors produced relatively little PSA and that PSA levels lost their correlation with PCa aggressiveness [25-28]. Moreover, in CRPC patients, the serum PSA levels are far behind the progression of PCa [26-28]. In our retrospective study of 40 cases of PCa (GS ≥ 6, serum PSA > 4 ng/ml), the AKR1C3 expression level exhibited a positive correlation with the GS (score from 6 to 9) and a negative correlation with PSA levels. Although the correlation index is low in this study, the data still indicate that the expression of AKR1C3 may serve as a promising biomarker for evaluating prostate cancer progression.

## Conclusions

Overexpressed AKR1C3, as an adaptive response for the progression of PCa, exhibited a positive correlation with the GS. Our study shed light on the potential of AKR1C3 to serve as a promising biomarker for the progression of PCa.

## Competing interests

The authors declare that they have no competing interests.

## Authors’ contributions

YT, Ljing Zhao and CX carried out most of experiments, participated in the design of the study, performed the statistical analysis, and drafted the manuscript. HZ, Ljuan Z, YL and JL helped to edit the paper. All authors have read and approved the final manuscript.
